# Assessing spatial variability in land-use impacts on river water quality: a case study of the yura river watershed, Japan

**DOI:** 10.1007/s00267-025-02269-0

**Published:** 2025-08-30

**Authors:** Minori Tokito, Satoshi Asano, Keitaro Fukushima, Kenta Watanabe, Izuru Saizen

**Affiliations:** 1 Graduate School of Agriculture, Kyoto University; Kitashirakawa Oiwake-cho, Sakyo-ku, Kyoto, 606-8502 Japan; 2 Graduate School of Global Environmental Studies, Kyoto University; Yoshida-honmachi, Sakyo-ku, Kyoto, 606-8501 Japan; 3 Faculty of Food and Agricultural Sciences, Fukushima University; 1, Kanayagawa, Fukushima, 960-1296 Japan; 4 Coastal and Estuarine Environment Research Group, Port and Airport Research Institute, 3-1-1 Nagase, Yokosuka, Kanagawa 239-0826 Japan

**Keywords:** river water quality, land-use impact, geographically weighted regression (GWR), watershed management, spatial variability, yura river watershed

## Abstract

This study presents a novel approach to investigating the spatial relationship between land use and river water quality by applying Geographically Weighted Regression (GWR), which explicitly accounts for the nested structure of sub-watersheds—a factor that has been frequently overlooked in previous studies. The Yura River watershed in Japan was selected as the study site, and electrical conductivity (EC) was used as a comprehensive indicator of water quality. To reflect local land-use impacts, we introduced the difference in EC between upstream and downstream sampling points (ΔEC) and allocated it to individual sub-watershed polygons. By analyzing both irrigation and non-irrigation seasons, the study found that key land-use types, such as paddy fields, water bodies, and evergreen broadleaved forests, exert varying influences on water quality depending on the season and location. The GWR model outperformed global regression models in capturing spatial heterogeneity and reduced residual spatial autocorrelation, thereby validating its effectiveness in watershed-scale environmental analysis. Importantly, this study is the first to integrate GWR with ΔEC while considering the hierarchical structure of sub-watersheds. This framework enables more accurate identification of localized land-use effects on water quality, which are often masked in global models. The findings underscore the need for region-specific land-use management and offer methodological insights for improving watershed conservation strategies in heterogeneous landscapes. By highlighting both seasonal variation and spatial dependency, this study provides a useful toolset for environmental monitoring and supports the development of targeted, evidence-based watershed policies.

## Introduction

In ecosystems that span forests to the sea, various substances are transported through running waters, such as river systems. Rainfall in mountain forests cascades downward, carrying mineral-rich soil into rivers and eventually to the sea, where it nourishes plankton that sustain marine life. This intricate water-based web of connectivity between forests, land, and seas represents complex ecological interactions that are closely intertwined with human activities and lifestyles (Meybeck 2003).

Since river systems are closely linked to their catchments, changes in human activities and lifestyles, such as land use within the watershed, directly and significantly impact the downstream environment (Bricker and Jones 1995; de la Cretaz and Barten 2007). For example, Malmqvist and Rundle (2002) noted that issues such as downstream eutrophication and coastal hypoxia result from upstream land-use changes and cumulative agricultural practices that increase nutrient runoff. River systems have long been a focus of environmental studies, as they serve as unidirectional transport systems, carrying significant dissolved and particulate material loads from natural and anthropogenic sources.

Numerous studies have demonstrated the relationship between river water quality and land use. Accumulated scientific evidence confirms that land use directly impacts river water quality (Basnyat et al. 1999; Jarvie et al. 2002; Tong and Chen 2002; Little et al. 2003; Woli et al. 2004; Mehaffey et al. 2005; Williams et al. 2005; Rodriguez et al. 2007). However, most of these studies did not consider spatial variations within watersheds. For instance, Lavergne et al. (2022) demonstrated how watershed land use affects the coastal environment and its biodiversity by correlating the richness of the most vulnerable estuarine fish species with watershed-scale land-use factors. The study found that species richness was higher where forest cover was most abundant, concluding that forest cover contributes to species conservation. This valuable result underscores the link between nature and human activities on a large watershed scale; however, it was based on a global regression model and did not account for spatial variations of land-use effects on water quality within the watershed. Therefore, the mechanisms and spatial variability of land-use effects on ecological systems in watersheds remain poorly understood.

Catchments are heterogeneous at all scales due to variability in factors such as precipitation, snowmelt, geology (material, bedrock topography, fractures), soil (matrix, macropores, soil moisture, thermal status, layering), vegetation, and land use (Beven et al. 1988; McDonnell et al. 2007). The quality of surface water flowing from upstream to downstream varies spatially, and land use affects water quality at different points (Simeonov et al. 2003), highlighting the importance of spatial differences. Although previous studies have developed hydrological models to identify general trends, none have accounted for the spatial differences in factors influencing water quality within a watershed. Tetzlaff et al. (2010) pointed out that the fundamental difficulties in understanding, managing, and protecting watershed ecosystems arise from the fact that most available information about relevant processes is derived from small-scale plots, hillslopes, and headwater catchments, which limits the applicability of such data at larger scales. In contrast, management and environmental issues that must be resolved typically occur at the landscape or regional scale. To develop effective regional plans for solving environmental problems, it is crucial to clarify the spatial differences in impacts within a watershed rather than relying solely on general trends at the global scale.

Several barriers hinder the clarification of spatial differences in impacts within a watershed. Many studies highlight the complex and hierarchical relationships among the components of stream water quality as a major challenge in spatially understanding the hydrological behavior of stream water quality (Beven et al. 1988; Band et al. 2000). Small watersheds are interconnected with larger ones, forming a continuous and spatially hierarchical structure; therefore, each small watershed is not independent. Failing to account for this hierarchical structure when evaluating the impact on water quality at a measurement point may result in misestimating or underestimating human impacts on river water quality. To overcome this issue, Uchida et al. (2005) examined the spatial pattern of river solute concentrations within a steep mountain watershed with a homogeneous landscape, and they were able to minimize the influence of landscape components, such as land use, clarify the relationship between watershed area and solute concentrations, and exclude the confounding effects of landscape unit and characteristic changes. Consequently, the influence of landscape elements, including land use, was systematically removed by Uchida et al. (2005). However, in watersheds with complex landscape elements, identifying factors that affect water quality is challenging due to the hierarchical structure of the watershed. Moreover, spatial differences within watersheds have not been clarified.

To address the limitations of previous studies that employed global regression models and overlooked spatial heterogeneity, this study uses Geographically Weighted Regression (GWR) as a novel analytical framework. GWR allows regression coefficients to vary across space (Fotheringham et al. 2002), enabling the detection and visualization of localized relationships between land use and water quality. Unlike traditional models, this spatially explicit approach offers a significant advantage by revealing how the strength and direction of land-use impacts on water quality differ within a watershed. While past studies have established general associations between land use and water quality, few have examined these relationships at finer spatial resolutions. Although GWR has been increasingly applied in diverse fields to identify spatially varying relationships, its use in watershed-scale analyses remains limited, particularly in the context of nested sub-watershed structures. By applying GWR to sub-watershed units and using electrical conductivity (EC) as a comprehensive water quality indicator, this study aims to clarify the spatial heterogeneity of land-use impacts on river systems and contribute to a more nuanced understanding of human–environment interactions within watersheds.

## Materials and methods

To examine the spatial heterogeneity of land-use impacts on river water quality, we conducted a watershed-scale analysis in the Yura River watershed, Japan. The analysis focused on EC as a comprehensive indicator of water quality and on high-resolution land use and land cover data derived from satellite imagery. We applied GWR to capture spatially varying relationships and, for comparison, also employed a Generalized Linear Model (GLM) to estimate global relationships. The following sections describe the study area, datasets, and analytical procedures in detail.

### Study site

**Fig. 1:**
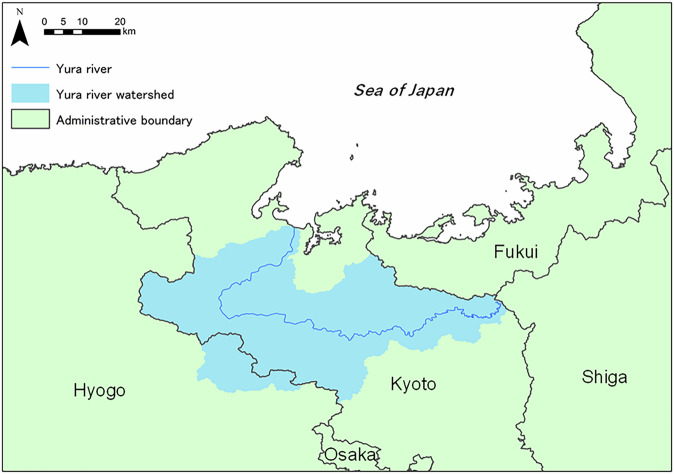
Study site

We selected the Yura River watershed as the study site, which is located in parts of Kyoto and Hyogo Prefectures, Japan, and the river flows into the Sea of Japan (Fig. 1). The river has a total length of 146 km and a catchment area of 1,880 km^2^, 89% of which is covered by forests. The riverbed slope (1/188) is steep, resulting in a short retention time for river water and relatively clear water with low humic content. The river discharge (annual mean < 50 m^3^ s^−^¹) is highest from winter to spring (February to April) due to snowmelt and from May to July due to high precipitation. In contrast, the discharge is generally low from August to January (Watanabe et al. 2017). The variations in the river discharge and water quality regulate nutrient supply into the coastal waters, affecting biological production there (Watanabe et al. 2017, 2018).

### Water quality indicator

EC refers to the ability of water to conduct current, which is primarily determined by the concentration of dissolved electrolytes. EC generally changes proportionally to the amount of electrolytes present in the water (Dojlido and Best 1993; Welch et al. 1998). A lower EC value indicates fewer impurities, while a higher EC value corresponds to a greater concentration of dissolved electrolytes.

As water flows across rocks and through watersheds, it can dissolve various substances, picking up dissolved and suspended ions such as sodium, potassium, chloride, carbonate, sulfate, calcium, and magnesium. These electrolytes originate from both natural influences, such as geology, topography, and meteorological conditions, and anthropogenic sources, such as wastewater from industrial and agricultural areas. The presence of these substances significantly alters water conductivity. EC is sensitive to changes in the concentration of electrolytes, making it a comprehensive indicator of ion dissolution from various land uses within a watershed (Cano-Paoli et al. 2019).

Although river water conductivity can be influenced by factors like watershed geology, watershed size, wastewater from point sources, runoff from non-point sources, atmospheric input, evaporation rates, and certain bacterial metabolism (Allan and Castillo 2007; Likens and Bormann 1995), EC is generally considered to increase from upstream to downstream (Kaushal et al. 2005). In the context of river conservation and management, the EC value tends to be higher downstream than upstream. This suggests that the location of the outflow point and the order of water flow within a watershed can help identify the factors influencing EC levels.

Despite its importance, the use of EC as a single water quality indicator has not been widely discussed in the literature. This may be because incorporating multiple chemical parameters can provide more comprehensive data. However, normalizing other physico-chemical parameters allows EC to serve as a potential standalone indicator for water quality pollution studies (Yap 2013). As an absolute value, EC can be directly incorporated into spatial analysis frameworks such as GWR, a feature not readily achievable with ionic composition data expressed in relative terms. This makes EC a practical and robust variable for exploring spatial heterogeneity in the relationship between land use and water quality. Therefore, in this study, we adopted EC as a comprehensive indicator when examining the spatial impacts of land use on water quality.

**Fig. 2:**
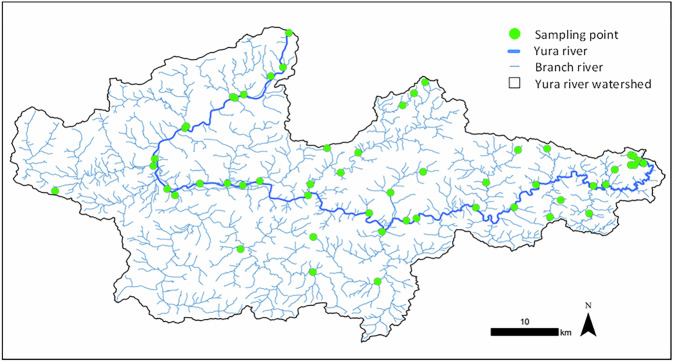
Yura River watershed and survey points

Water-quality data were collected at 54 sites along the Yura River watershed (Fig. 2). We used data collected in 2011, when sampling within the watershed covered the largest number of sites and the widest spatial extent. Field surveys were conducted from April 25th to 28th, 2011 (irrigation season) and from October 31st to November 2nd, 2011 (non-irrigation season). Salinity was measured in situ for each water sample using an electrical conductivity meter (SC72; Yokogawa Electric, Tokyo, Japan).

Each sub-watershed was defined as the catchment area corresponding to one of the 54 sampling sites. The sub-watersheds were delineated using the Create Watersheds tool in ArcGIS 10.8.1 (ESRI, Redlands, CA, USA), with each sampling point designated as a pour point. This tool identifies the upstream contributing area for each pour point based on flow direction and accumulation derived from a digital elevation model (DEM). This process ensured a one-to-one correspondence between each sampling location and its associated sub-watershed.

**Fig. 3:**
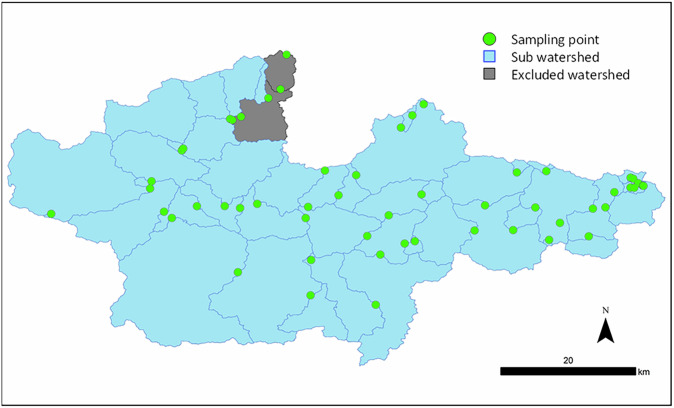
Sub watershed and excluded area

Note that two sub-watershed polygons nearest the mouth of the river were excluded from the analysis (Fig. 3). The downstream portion of the Yura River is classified as a salt-wedge estuary with a spring tidal range of less than 0.5 m. The salt wedge can extend up to 20 km upstream during low river discharge (Kasai et al. 2010; Watanabe et al. 2014). Tidal fluctuations cause the saltwater to enter and exit the estuary, resulting in variations in water quality parameters, including conductivity and salinity. To eliminate the influence of salt-wedge intrusion, these two sub-watershed polygons were excluded.

**Fig. 4:**
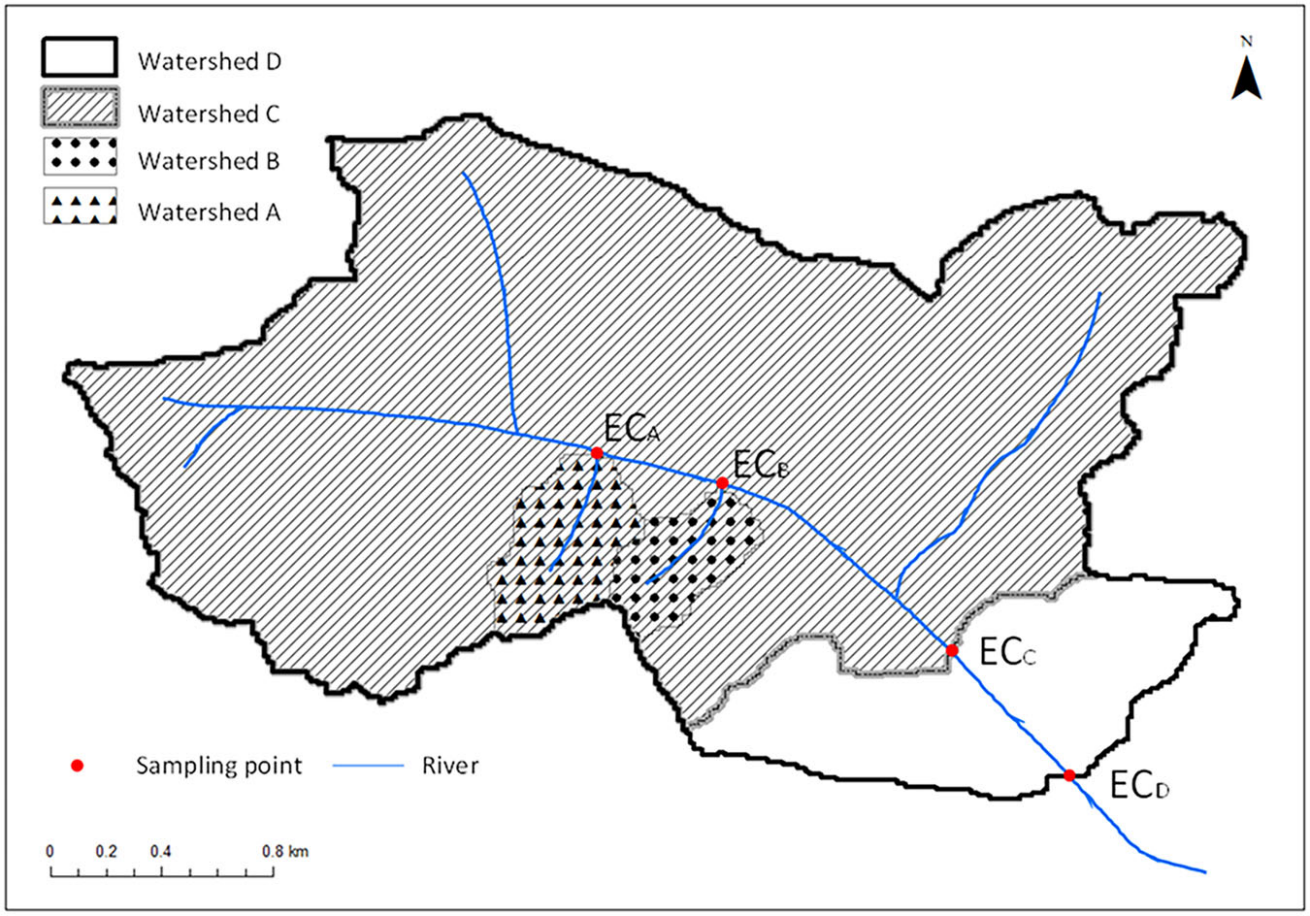
Example for $\Delta {EC}$ calculation

To account for the nested structure of sub-watersheds, we calculated the delta of electrical conductivity ($\Delta {EC}$) between the upper and lower sub-watersheds and allocated these values to each sub-watershed polygon. $\Delta {EC}$ can be calculated as follows.

1 $$\left({{EC}}_{l}\right)-\left({{EC}}_{u}\right)=\Delta {EC}$$

where ${{EC}}_{l}$ is the EC at the lower sampling site and ${{EC}}_{u}$ is the EC at the upper sampling site. For example, in Fig. 4, $\Delta {{EC}}_{A}$ equals ${{EC}}_{A}$ since watershed A is the highest part of the watershed. Similarly, $\Delta {{EC}}_{B}$ equals ${{EC}}_{B}$. However, watershed C, located lower than watersheds A and B, requires adjustment for the nested structure. Thus, $\Delta {{EC}}_{C}$ is calculated by applying the differences in EC, weighted by the watershed area ratio. The $\Delta {EC}$ values are calculated as follows:

2 $$\Delta {{EC}}_{A}={{EC}}_{A}$$

3 $$\Delta {{EC}}_{B}={{EC}}_{B}$$

4 $$\Delta {{EC}}_{c}={{EC}}_{C}-\left(\frac{{{EC}}_{A}{S}_{A}+{{EC}}_{B}{S}_{B}}{{S}_{C}}\right)$$

5 $$\Delta {{EC}}_{D}={{EC}}_{D}-\left(\frac{{{EC}}_{C}{S}_{C}}{{S}_{D}}\right)$$

where $S$ represents the area of the watershed.

The $\Delta {EC}$ values, defined and calculated above, were used as indicators in the analysis. $\Delta {EC}$ values from both irrigated and non-irrigated seasons were employed as the dependent variable.

### Land use indicators

High-resolution land use and land cover map data were used to explore the spatial relationships between land use and water quality. These data were obtained from the Advanced Visible and Near Infrared Radiometer type 2 (AVNIR-2) onboard the Japanese satellite “Daichi” (ALOS) and provided by the Earth Observation Research Center (EORC) of the Japan Aerospace Exploration Agency (JAXA). The dataset is an open-access, composite product representing land-use and land-cover conditions between 2006 and 2011, created by integrating multi-year AVNIR-2 observations; accordingly, it reflects average conditions over that period rather than a single time point. The map had a mesh size with a 1/12,000-degree resolution, corresponding to approximately 10-meter square grid cells. Although the water-quality surveys were conducted in 2011, this composite spans the sampling year and provides a temporally averaged, stable baseline for the spatial analysis by reducing short-term variability and data gaps.

**Fig. 5:**
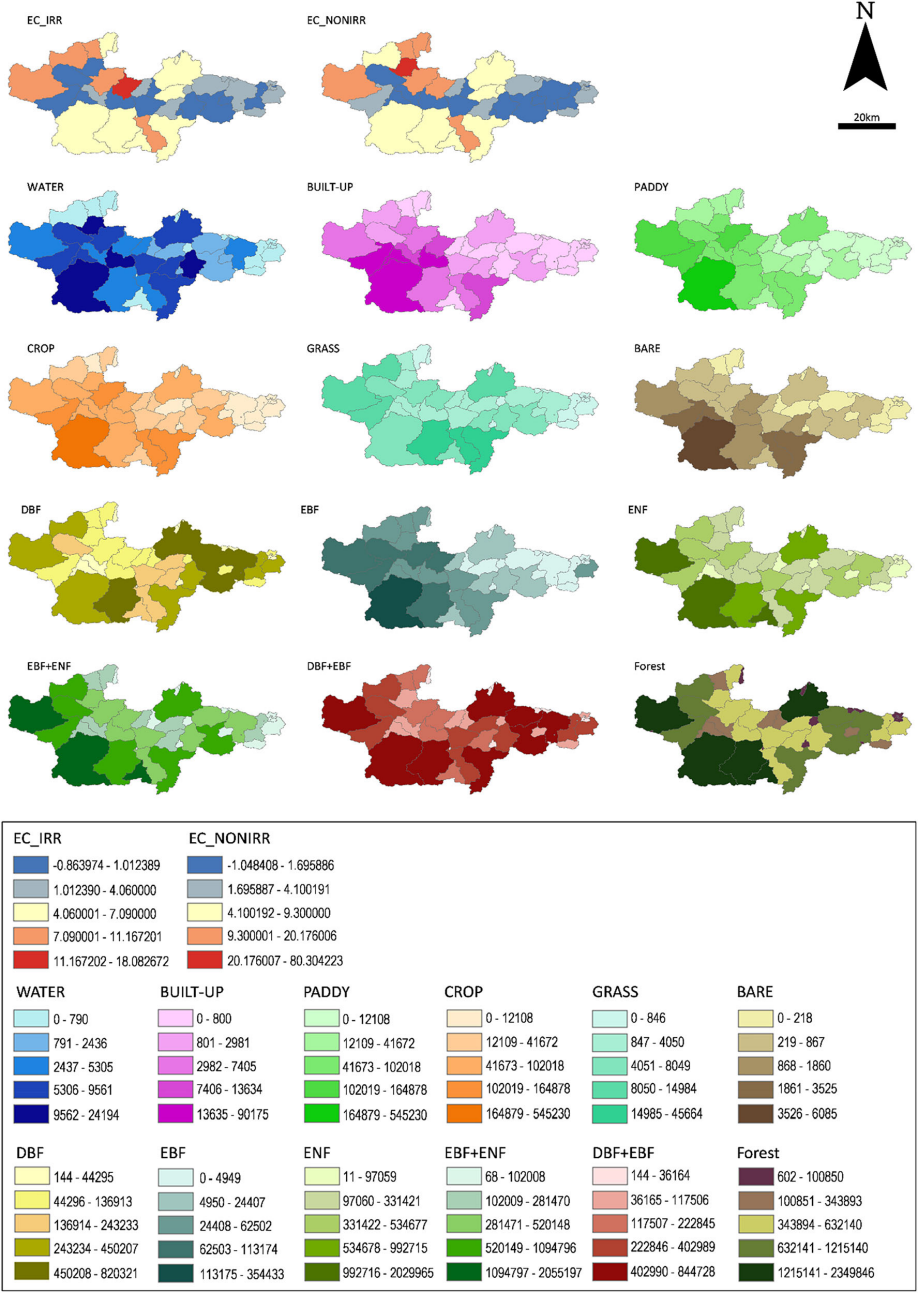
Maps of the dependent and independent variables

The number of grids in nine land cover categories (Water bodies, Built-up area, Paddy field, Cropland, Grassland, Bare land, Deciduous broadleaved forest; DBF, Evergreen broadleaved forest; EBF, and Evergreen needle-leaf forest; ENF) was selected as the independent variable. These categories were derived from the high-resolution land use and land cover map data (2006–2011) provided by JAXA. To account for variations in forest types, we created three new categories for forest types: number of grids of Evergreen Forest (EBF + ENF), number of grids of Broadleaved Forest (DBF + EBF), and number of grids of Forest (DBF + EBF + ENF). Table 1 presents the dependent (Y) and independent (X) variables used in the model, with corresponding data shown in Fig. 5. Table 2 provides the subdivided categories based on forest types.

**Table 1:** Dependent (Y) and independent (X) variables used in the GWR model

Variable	Variable Code	Detail Explanation
${Y}_{1}$	EC_IRR	$\Delta {EC}$ of irrigation season (data collected from April 25th to 28th, 2011)
${Y}_{2}$	EC_NONIRR	$\Delta {EC}$ of non- irrigation season (data collected from October 31st to November 2nd, 2011)
${X}_{1}$	WATER	Number of grids of Water bodies
${X}_{2}$	BUILT-UP	Number of grids of Built-up area
${X}_{3}$	PADDY	Number of grids of Paddy field
${X}_{4}$	CROP	Number of grids of Cropland
${X}_{5}$	GRASS	Number of grids of Grassland
${X}_{6}$	BARE	Number of grids of Bare land
${X}_{7}$	DBF^*^	Number of grids of Deciduous broadleaved forest
${X}_{8}$	EBF^*^	Number of grids of Evergreen broadleaved forest
${X}_{9}$	ENF^*^	Number of grids of Evergreen needle-leaf forest
${X}_{10}$	EBF + ENF^*^	Number of grids of Evergreen forest
${X}_{11}$	DBF + EBF^*^	Number of grids of Broadleaved forest
${X}_{12}$	FOREST^*^	Number of grids of Forest (DBF + EBF + ENF)

^*^ Variables related to forests (*X*_*7*_ – *X*_*12*_) should be selected depending on the subdivided categories (Table 2)

**Table 2:** Category subdivisions based on the forest type

All types	Deciduous/Evergreen	Broadleaved/Coniferous	No division
DBF	DBF	DBF + EBF	FOREST
EBF	EBF + ENF		
ENF		ENF	

### Modeling approach

#### Geographically weighted regression for analyzing spatial variations in land use and water quality

To explore the spatial variations in the relationship between land use and water quality, we employed two regression models: Generalized Linear Model (GLM) and Geographically Weighted Regression (GWR). GLM assumes spatial stationarity and produces a single global estimate for each parameter, while GWR allows model coefficients to vary by location, capturing localized spatial dependencies (Fotheringham et al. 2002). The formula for the GWR model is as follows:

6 $${Y}_{i}={\beta }_{0}\left({u}_{i},{v}_{i}\right)+\mathop{\sum }\limits_{j=1}^{p}{\beta }_{j}\left({u}_{i},{v}_{i}\right){X}_{{ij}}+{\varepsilon }_{i}$$

where ${Y}_{i}$ is the dependent variable for observation $i$, ${X}_{{ij}}$ is the value of the $j$-th independent variable for observation $i$, and $\left({u}_{i},{v}_{i}\right)$ are the spatial coordinates of observation $i$. ${\beta }_{0}\left({u}_{i},{v}_{i}\right)$ is the local intercept, ${\beta }_{j}\left({u}_{i},{v}_{i}\right)$ is the local regression coefficient, and ${\varepsilon }_{i}$ is the random error term. This approach enables the estimation of local parameter values, revealing spatial heterogeneity in the relationships between the dependent and independent variables.

GWR has been applied in a wide range of studies to identify spatial heterogeneity in social, ecological, and urban systems (Farrow et al. 2005; Shi et al. 2006; Yu 2006). Tu and Xia (2008) applied GWR to analyze the relationship between land use and water quality in eastern Massachusetts, USA. However, their study did not account for the nested structure of small watersheds. To explore spatial relationships between land use and water quality using GWR analysis, it is necessary to address the nested structure of sub-watersheds and independently capture each unit.

In this study, GWR offers a methodological advantage by allowing us to assess how the strength and direction of land-use impacts on water quality differ across sub-watersheds. Unlike prior applications (Tu and Xia 2008), we explicitly considered the nested structure of the sub-watersheds in our modeling process.

#### Model comparison and selection criteria

This study investigated the impact of land use on water quality using two regression models, the GLM and GWR models, both implemented via the R package (Gollini et al. 2015). GLM estimates a single set of global regression coefficients applicable to all observations, while GWR accounts for spatial heterogeneity by estimating local regression coefficients at each geographic location. We aimed to compare the results of the GWR and GLM models to identify which model better captured spatial variations in the relationship between land use and water quality.

To achieve this, we constructed models using all possible combinations of independent variables for each dependent variable (Y1 and Y2). For forest cover-related variables (X₇–X₁₂), we selected variables based on their respective subcategories to avoid redundancy. This process resulted in the development of 831 GLM and 831 GWR models for each dependent variable. The optimal bandwidth for GWR was determined by minimizing either the cross-validation (CV) or the corrected Akaike Information Criterion (AICc) score. We evaluated both the CV and AICc methods for all models and selected the best model based on a comprehensive evaluation, which included model performance (AICc, adjusted R^2^), multicollinearity among variables, and the presence of spatial autocorrelation. Based on the results of this evaluation, different selection criteria were ultimately applied for the irrigation and non-irrigation periods, thus ensuring that the chosen bandwidth provided the most reliable and interpretable model for each period.

Model performance was assessed using the AICc and adjusted R^2^ metrics (Miller 2012). Adjusted R^2^ indicates the proportion of variance in the dependent variable explained by the independent variable, with higher values preferred. AICc is a measure of model fit, with smaller values indicating better performance. Additionally, we evaluated the spatial distribution of residuals from the selected model using spatial autocorrelation analysis (Global Moran’s I). If the residuals exhibited spatial clustering, this suggested that key independent variables might have been omitted from the model.

By comparing the performance of the GWR and GLM models, we identified the model that better captured spatial variations in the relationship between land use and water quality. Finally, we generated spatial maps of the estimated parameters from the selected model to visualize the spatially varying relationships between land use and river water quality.

## Result

### Relationships between land use and water quality of the irrigation season

We first examined the land-use factors influencing the dependent variable Y1 ($\Delta {EC}$ of the irrigation season). After evaluating 831 models with different combinations of independent variables, the lowest AICc values were obtained when using “WATER” and “PADDY” as predictors.

Table 3 summarizes the model performances for both the GLM and GWR models. The AICc values for the GLM and GWR models were 264.45 and 249.74, respectively, indicating a better fit for the GWR model. The adjusted R^2^ was also higher for the GWR model (0.52) than for the GLM model (0.27), suggesting that GWR explained more variance in the response variable. Additionally, spatial autocorrelation in the residuals was significant in the GLM model (Moran’s I = 0.33, *p* < 0.001), whereas the GWR model substantially reduced it (Moran’s I = 0.13, *p* = 0.06). The non-significant p-value in the GWR model suggests that the residuals did not exhibit spatial clustering, confirming that GWR effectively accounted for spatial heterogeneity. These results imply that the selected independent variables played a significant role in explaining the variation in Y1, with minimal indication of missing key factors.

**Table 3:** Summary of model performances for GLM and GWR models

Model	Diagnostic information				Spatial Autocorrelation	
	AIC	AICc	BIC	Adjusted R2	Moran’s I statistic	*P* value
GLM	263.58	264.45	236.03	0.27	0.33	<0.001
GWR	231.23	249.74	209.22	0.52	0.13	0.06

In the GLM results (Table 4), both WATER and PADDY were statistically significant. WATER exhibited a negative effect (Estimate = −0.00, *p* < 0.001), meaning that an increase in WATER was associated with a decrease in Y1. Conversely, PADDY had a positive effect (Estimate = 0.00, *p* < 0.001), indicating that greater PADDY values corresponded to an increase in Y1. The Variance Inflation Factor (VIF) values were ~2.60, which suggested no serious multicollinearity issues, with values above 5 typically indicating concern.

**Table 4:** Coefficient estimates for the explanatory variables in the global regression model (irrigation season)

	Estimate	Std. Error	*t* value	Pr(>|t | )	VIF
Intercept	4.37	0.52	8.48	<0.001***	-
WATER	−0.00060	0.00015	−4.09	<0.001***	2.60
PADDY	0.000037	0.0000083	4.44	<0.001***	2.60

Signif. codes: 0 ‘***‘ 0.001 ‘**‘ 0.01 ‘*‘ 0.05 ‘.’ 0.1 ‘ ‘ 1

**Fig. 6:**
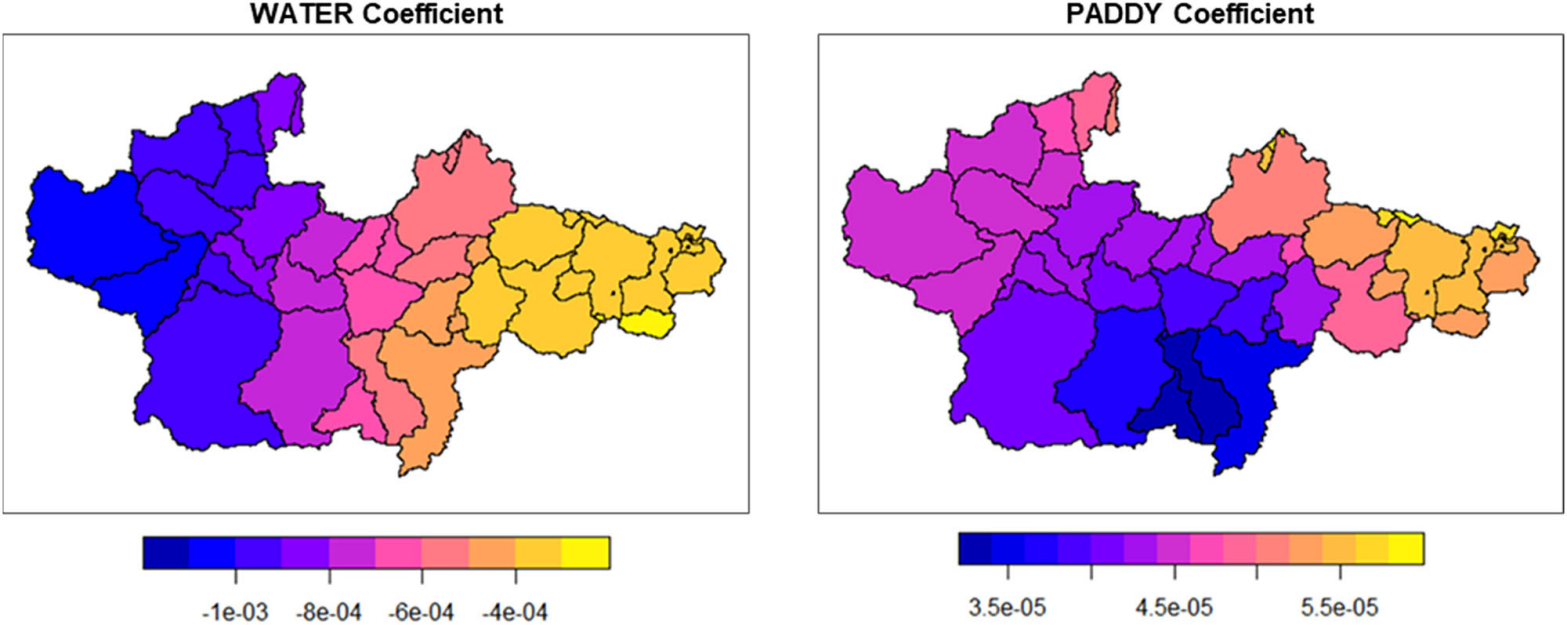
Spatial distribution maps of the parameter estimate (irrigation season)

The GWR model, which provided a better fit than the GLM model, revealed notable spatial variability in the impact of WATER but not PADDY. Table 5 summarizes the results of the GWR model, and Fig. 6 illustrates the spatial distribution of the local coefficient estimates. For WATER, the coefficients ranged from −1.36E-03 to −2.00E-04, consistently showing negative effects but varying in magnitude across locations. For PADDY, the coefficients ranged from 3.81E-06 to 1.00E-04, with relatively stable effects across space. The significant F3 statistic for WATER (3.82, p < 0.001) confirmed its spatial variability, whereas PADDY remained spatially stable (F3 = 0.73, p = 0.55). This suggests that the impact of WATER on Y1 varied considerably depending on location, while the influence of PADDY was more uniform across the study area.

**Table 5:** Coefficient estimates for explanatory variables in the GWR model (irrigation season)

	Min.	1st Qu.	Median	3rd Qu.	Max.	F3 statistic	Pr(>)
Intercept	2.34E + 00	2.65E + 00	3.92E + 00	6.41E + 00	7.64	7.98	<0.001***
WATER	−1.36E-03	−9.10E-04	−5.79E-04	−3.18E-04	−0.0002	3.82	<0.001***
PADDY	3.81E-06	3.71E-05	4.82E-05	5.86E-05	0.0001	0.73	0.55

The optimal bandwidth was 10280.15 m, as determined by minimizing CV score

Signif. codes: 0 ‘***‘ 0.001 ‘**‘ 0.01 ‘*‘ 0.05 ‘.’ 0.1 ‘ ‘ 1

### Relationships between land use and water quality of the non-irrigation season

We examined the impact of land-use factors on the dependent variable Y2 ($\Delta {EC}$ during the non-irrigation season). After evaluating 831 models with various combinations of independent variables, the lowest AICc values were observed when “WATER” and “EBF” were used as predictors.

Table 6 presents the model performance comparisons for both GLM and GWR. The AICc value for the GWR model (356.27) was substantially lower than that for the GLM model (398.12), confirming the superiority of GWR. Furthermore, the GWR model demonstrated a significantly better fit (Adjusted R^2^ = 0.84) compared to the GLM (Adjusted R^2^ = 0.02), highlighting its effectiveness in explaining the variance in Y2. The Moran’s I test revealed that the residuals in the GLM exhibited significant spatial autocorrelation (Moran’s I = 0.33, p < 0.001), while the GWR model effectively reduced this effect (Moran’s I = 0.09, p = 0.14). These results suggest that GWR successfully captured spatial heterogeneity, making it the more appropriate model for this analysis.

**Table 6:** Summary of model performances for GLM and GWR models (non-irrigation season)

Model	Diagnostic information				Spatial Autocorrelation	
	AIC	AICc	BIC	Adjusted R2	Moran’s I statistic	*P* value
GLM	397.25	398.12	369.71	0.015	0.33	< 0.001
GWR	277.02	356.27	296.89	0.84	0.09	0.14

The GLM results (Table 7), indicated that neither WATER nor EBF were statistically significant, implying that their effects are spatially dependent and not adequately represented in a global model. The Variance Inflation Factor (VIF) value of 2.468856 indicated no concerns with multicollinearity. However, the GWR model revealed substantial spatial variability in the effects of both WATER and EBF, confirming the presence of spatial heterogeneity. The F3 statistic for both variables was significant (*p* < 0.001), further justifying the use of GWR over GLM. This confirms that applying GWR allowed us to capture spatially variable relationships that the global model could not.

**Table 7:** Coefficient estimates for the explanatory variables in the global regression model (non-irrigation season)

	Estimate	Std. Error	*t* value	Pr(>|t | )	VIF
Intercept	5.06E + 00	1.91E + 00	2.65	0.01*	–
WATER	7.57E-04	5.31E-04	1.43	0.16	2.47
EBF	−2.46E-05	4.47E-05	−0.55	0.58	2.47

Signif. codes: 0 ‘***‘ 0.001 ‘**‘ 0.01 ‘*‘ 0.05 ‘.’ 0.1 ‘ ‘ 1

**Fig. 7:**
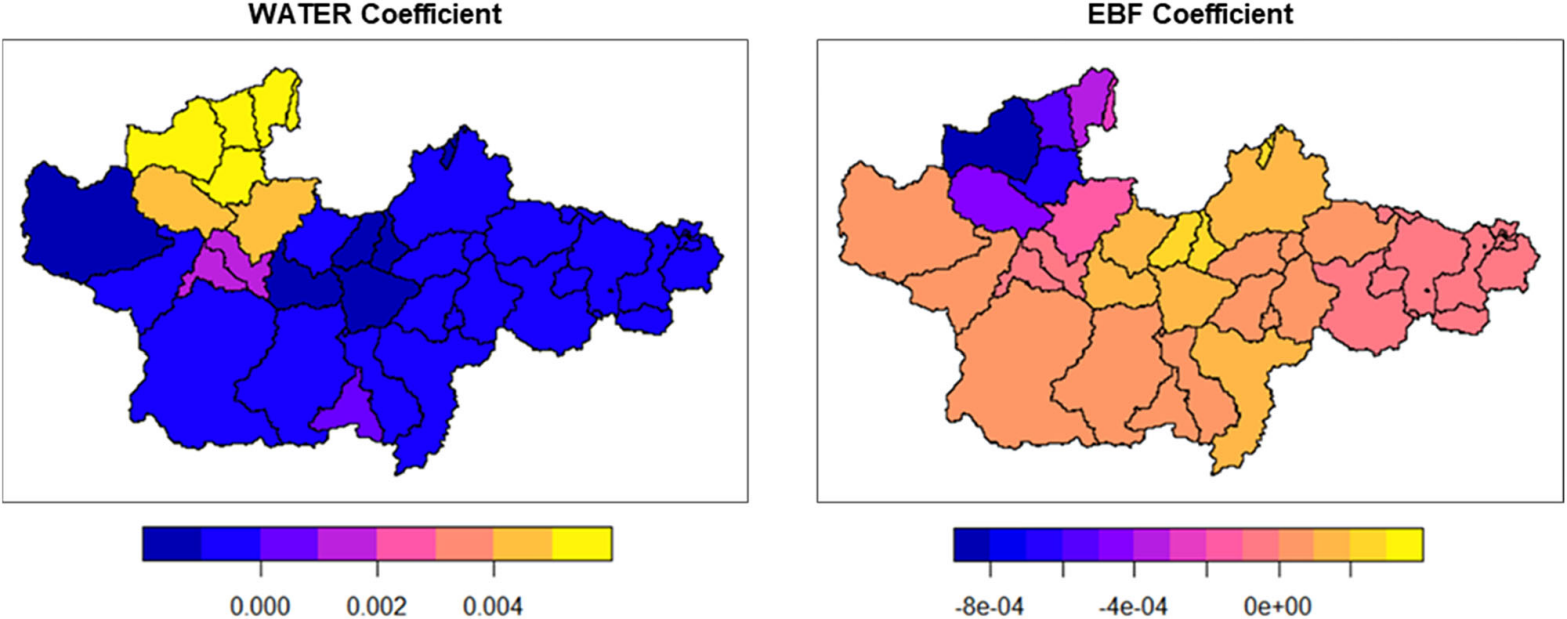
Spatial distribution maps of the parameter estimate (non-irrigation season)

Figure 7 illustrates the spatial distribution of local coefficient estimates, revealing that the effect of WATER ranged from −1.92E-03 to 0.0059 across different regions (Table 8). This variability suggests that WATER’s influence is not consistent and may exhibit opposing effects depending on location.

**Table 8:** Coefficient estimates for explanatory variables in the GWR model (non-irrigation season)

	Min.	1st Qu.	Median	3rd Qu.	Max.	F3 statistic	Pr(>)
Intercept	−4.66E + 00	3.02E + 00	4.91E + 00	7.06E + 00	48.45	9.78	< 0.001 ***
WATER	−1.92E-03	−8.02E-04	−5.54E-04	−2.27E-04	0.0059	7.24	< 0.001 ***
EBF	−8.99E-04	−1.29E-05	−4.49E-06	8.01E-05	0.0003	4.36	< 0.001 ***

The optimal bandwidth was 5186.566 m, as determined by minimizing AICc score

Signif. codes: 0 ‘***‘ 0.001 ‘**‘ 0.01 ‘*‘ 0.05 ‘.’ 0.1 ‘ ‘ 1

## Discussion

As a result of the modeling, different combinations of independent variables were adopted for the irrigation and non-irrigation seasons. This variation indicates that the key factors influencing water quality differ between these seasons, thus reflecting the impact of seasonal environmental changes. For instance, Ko et al. (2021) investigated seasonal variations in water quality and biodiversity in the Yasu River, a tributary of Lake Biwa in Japan, and clarified how seasonal changes in physical and chemical factors affect the aquatic environment. The findings of this study align with these observations, particularly highlighting that changes in ΔEC effectively capture water quality variations between the irrigation and non-irrigation seasons.

### Relationship between water quality variations and land use during the irrigation season

Ko et al. (2021) demonstrated that during the irrigation season, drainage from paddy fields alters river water quality and affects biodiversity. Similarly, this study identified PADDY as a key variable influencing water quality during the irrigation season, suggesting that irrigation drainage from paddy fields impacts river water quality. The relationship between irrigation and water quality during the irrigation season is well-documented in previous studies. For example, Sakai et al. (2013), Okano et al. (2018), and Ishida et al. (2020) reported that paddy field irrigation contributes to eutrophication and sedimentation in receiving rivers and coastal areas through the discharge of dissolved and particulate nutrients. Moreover, Ishikawa and Zhang (2016) examined the effects of fine sediment runoff from paddy fields on river turbidity in the Egawa River watershed in northeastern Japan, finding that sediment runoff during farming activities, such as puddling and mid-season drainage, contributes to increased river turbidity.

The results of this study, which applied GLM and GWR by treating sub-watersheds as independent polygons using ΔEC, are consistent with previous findings on the impact of paddy field irrigation. This consistency suggests that the methodology used in this study effectively explains the relationship between water quality variations and land use during the irrigation season. However, in the case of ΔEC during the irrigation season, both WATER and PADDY were significant in the GLM model, but only WATER exhibited spatial variation in the GWR model (F3 test: p < 0.001), while PADDY showed no significant spatial variability (F3 test: p = 0.55). The coefficient for WATER ranged from −1.36E-03 to −2.00E-04 across the Yura River watershed, indicating that the strength of its negative impact varied by location, highlighting the importance of applying GWR. In contrast, the coefficient for PADDY ranged from 3.81E-06 to 1.00E-04, suggesting a positive impact with minimal regional variation. Therefore, GLM alone may provide an accurate estimate without the need for spatial methods such as GWR.

### Relationship between water quality variations and land use during the non-irrigation season

During the non-irrigation season, WATER and EBF were identified as key land-use factors influencing ΔEC. According to the GLM results, WATER had a positive effect on ΔEC, while EBF exhibited a negative effect. This contrasts with the irrigation season results, where WATER consistently had a negative impact on ΔEC. However, the GWR model for the non-irrigation season revealed spatial variation in the coefficient values of WATER, ranging from −1.92E-03 to 0.0059, suggesting regional differences in its influence. In three-quarters of all sub-watershed polygons, WATER had a positive effect, indicating that, in most areas of the Yura River watershed, an increase in water body area is associated with a decrease in ΔEC (Table 5). In contrast, in the downstream region, the coefficient for WATER was positive (0.0059), implying that an increase in water body area may lead to an increase in ΔEC (Fig. 6). The F3 test confirmed the statistical significance of this spatial variation, suggesting that the relationship between water body area and water quality is not uniform and may depend on watershed characteristics. For example, in downstream urban areas, an increase in water body area may lead to higher pollutant inflows, while in forested areas, dilution effects may dominate. Identifying these factors requires a more detailed analysis of land use and pollution sources at the watershed level.

Another significant factor influencing ΔEC during the non-irrigation season was EBF. The relationship between river water quality and forest cover has been extensively explored in previous studies. For example, Brogna et al. (2017) evaluated the relationship between forest cover and water quality in the Walloon region of Belgium, concluding that forest cover explained approximately one-third of water quality variations, with increased forest area positively correlated with water quality improvement. A meta-analysis by Qiu et al. (2023) also found that increased forest cover was associated with reduced total nitrogen and phosphorus concentrations, while forest fragmentation was linked to water quality deterioration. These findings reinforce the well-established role of forests in water resource conservation, such as water retention and soil erosion prevention, which likely contribute to the negative effect of EBF on ΔEC observed in this study’s GLM results. Notably, this study specifically identified EBF as an important variable influencing water quality.

Evergreen broadleaved trees possess thick cuticle layers that repel water (Kerstiens 1996), reducing the leaching of water-soluble substances when rainwater flows over their leaves (Levia and Frost 2006). Compared to deciduous broadleaf and coniferous trees, evergreen broadleaved trees have higher amounts of recalcitrant components like lignin and wax, leading to slower decomposition and reduced release of water-soluble substances (Aerts 1997; Cornwell et al. 2008). Additionally, evergreen broadleaved trees have dense foliage, which reduces direct rainfall penetration and prolongs surface water flow over leaves (Crockford and Richardson 2000; Keim et al. 2006). Although this can increase ion leaching from leaves, the cuticle layer may restrict leaching (Tukey 1970; Levia and Germer 2015). Furthermore, evergreen broadleaved trees generally exhibit lower stemflow volumes than other forest types, reducing the concentrated transport of water-soluble substances (Levia and Frost 2006). For example, leaves from deciduous trees fall simultaneously in autumn, leading to rapid decomposition and frequent leaching. In contrast, evergreen trees retain their leaves longer and gradually replace them, which results in a continuous but smaller release of water-soluble substances over time (Staelens et al. 2008). Based on these characteristics, the negative effect of evergreen broadleaved trees on ΔEC during the non-irrigation season, as observed in the GLM results, may reflect their tendency to suppress the leaching of water-soluble substances.

The GWR results for EBF showed significant spatial variability in coefficient values, ranging from −8.99E-04 to 0.0003 (F3 test: *p* < 0.001), with an overall negative effect but a positive effect in one-quarter of the sub-watershed polygons (Fig. 7). This suggests that, in certain locations, EBF may contribute to increased ΔEC. However, the absolute values of the EBF coefficients were generally small, indicating that its impact may be relatively minor compared to other factors.

### Need for appropriate land-use management in sub-watersheds

The results of the GWR model for the non-irrigation season revealed statistically significant spatial variations for both WATER and EBF, supporting the suitability of GWR in explaining the relationship between land use and water quality. Similarly, during the irrigation season, GWR detected spatial variation in the effect of WATER but not PADDY, which was inconsistent with the non-irrigation season. This discrepancy underscores the region-specific dynamics of water quality, as both models showed that land-use factors had either positive or negative effects on ΔEC depending on the location. These findings highlight the spatially varying impact of water bodies and forest areas on water quality, emphasizing the necessity for localized management approaches rather than uniform regional strategies.

In Japan, the decline in Satoyama management has led to secondary succession, with an increase in EBF (Takeuchi 2010). Historically, Satoyama landscapes were maintained by periodic harvesting of deciduous broadleaved trees, such as *Quercus serrata* and *Quercus acutissima*, for firewood and charcoal. These practices also control understory growth and prevent forest floor accumulation (Iida and Nakashizuka 1995; Itô et al. 2012). However, the abandonment of such management has allowed evergreen broadleaved species, such as *Castanopsis* and *Machilus*, to dominate, particularly in western Japan, including the Yura River watershed (Nakajima et al. 2018; Jiao et al. 2019). Given the increasing prevalence of evergreen broadleaved forests, the relationship between EBF and water quality identified in this study, though minor, should be recognized as a significant factor in the region’s evolving ecosystem dynamics.

### Limitations and future directions

One limitation of this study is that EC, as a measure of total ionic concentration, cannot differentiate among specific ion species. Different land-use types may cause EC increases through distinct ionic pathways, such as Ca^2+^ and SO_4_^2^− from geological sources (Tong and Chen, 2002), NO₃^−^ and Mg^2+^ from agricultural runoff (Woli et al. 2004), or Na^+^ and Cl^−^ from domestic wastewater (Cano-Paoli et al. 2019). While ΔEC effectively captures overall changes in ionic load, it does not reveal the specific mechanisms behind those changes, and should thus be regarded as an integrated indicator rather than a diagnostic one.

Despite this limitation, EC has a notable advantage—because it is expressed as an absolute value, it can be directly applied to spatial analysis frameworks such as GWR. In contrast, ionic composition is expressed in relative terms, making it unsuitable for the same type of spatial modeling. This makes ΔEC a practical and robust variable for exploring spatial heterogeneity in the relationship between land use and water quality, as demonstrated in this study.

Future research could address the limitation noted above by incorporating ionic composition profiles, such as the relative abundance of major ions, as supplementary information to aid interpretation. Overlaying GWR results with land-use-specific ion signatures may offer a way to infer the sources of conductivity variations and their spatial patterns. This integrative approach has the potential to enhance both scientific understanding and the practical management of watersheds.

## Conclusion

This study presents a novel framework that integrates Geographically Weighted Regression (GWR) with ΔEC, a spatially differentiated index based on electrical conductivity (EC), to investigate the relationship between land use and river water quality while explicitly accounting for the nested structure of sub-watersheds. By calculating ΔEC as the difference between upstream and downstream EC values and allocating it to sub-watershed polygons, we were able to reflect the directional flow and hierarchical connectivity of river systems. This approach enabled us to reveal spatial and seasonal variations in how land-use influences water quality.

The GWR results not only captured the spatial variability of the covariates but also provided valuable insights into the relative importance of local ecological drivers. Consequently, GWR emerges as a promising tool for identifying spatially varying ecological relationships and offering actionable insights for localized land-use management in heterogeneous landscapes.

## Data Availability

The water quality data used in this study were collected as part of the Research project of the connectivity of hills, humans and oceans on the local society based on ecosystem services in the forested watershed environment. These data are not publicly available due to project restrictions but may be available from the corresponding author upon reasonable request and with permission from the project lead.
